# Defining Disease in the Information Age

**DOI:** 10.1371/journal.pmed.0030317

**Published:** 2006-07-25

**Authors:** Olavo B Amaral

**Affiliations:** **1**Universidade Federal do Rio Grande do SulPorto AlegreBrazil

The series of disease mongering articles in the April 2006 issue of *PLoS Medicine* overall seem to define the term as “widening the boundaries of illness” [ 1] by “taking a normal function and implying that there's something wrong with it and that it should be treated” [ 2]. While there is undoubtedly a strong case to be made for this sort of practice by pharmaceutical companies, perhaps we should also question ourselves on what we mean by “disease boundaries.”

All of the conditions touched on by the disease mongering series (e.g., bipolar disease, attention deficit hyperactivity disorder, restless legs syndrome, and sexual dysfunctions) share the fact that they represent spectra of symptoms felt by virtually everyone, but which for some people can reach a point at which they become disturbing. However, since the benefit of treating these symptoms is ultimately dependent on their significance in a patient's life, it seems doubtful that anyone but the patient can adequately define the “boundaries” of illness for these conditions.

The existence of these large “grey zones” between disease and normality (as well as the difficulty of doctors in dealing with them) might help to explain the increase in “lifestyle drug use” and self-prescription of psychiatric medication [ 3]. While these behaviors undoubtedly carry risks, they might well be an inevitable development in an age where information on anything (including drugs) is so widely available. Moreover, tampering with body chemistry is nothing new (alcohol, coffee, chocolate, and sunlight come to mind as examples), and it is hard to expect people will not do it because of pharmaceutical labels. Therefore, complain as we may, it is unlikely that this trend can be feasibly prevented.

Therefore, if we want to prevent disease mongering, perhaps we should start by focusing on our own concept of “disease.” Maybe it is time we start to loosen the grip on our powers to define disease and start working less as diagnosing machines and more as decision facilitators for patients. It seems quite absurd to decide on a “concept” of erectile dysfunction or depression that can define who should be treated. On the contrary, our role should be to inform patients of the benefits and risks of treatment (or nontreatment) for their particular condition. This also means being comfortable with the fact that, no matter which criteria one uses to define disease, there will always be “normal” people who will want treatment as well as “sick” people who will refuse it. And in both cases they are probably entitled to do so, without necessarily receiving a diagnosis of “normal” or “sick.”

Moreover, since the trend for self-prescription is not likely to be prevented, and since the pharmaceutical industry will surely try to capitalize on it, perhaps we should also worry about making nonprofit, unbiased scientific information more available to the public. Education on health matters is an important responsibility that traditionally has been overlooked by doctors in most countries. Now, if ever, seems to be the time to change that, because if physicians do not concentrate on it, drug companies will be happy to do it for them.

It is obvious that medicine cannot abandon the concept of disease boundaries, since most of our medical knowledge and research is still based on it. Moreover, there are fields in which medical responsibility is sure to remain important in defining these boundaries (e.g., attribution of public funds, research studies, and treatment of children). But after reading so much on disease mongering, it seems to me that if we become a little more flexible in admitting that “disease boundaries” for many conditions are an oxymoron, perhaps the pharmaceutical industry will make less of a fuss in trying to convince people they are ill. My guess is that this would do everybody a favor.
